# Personalized surgical planning for coronary bypass graft configurations using patient-specific computational modeling to avoid flow competition in arterial grafts

**DOI:** 10.3389/fcvm.2023.1095678

**Published:** 2023-02-02

**Authors:** Krish Chaudhuri, Alexander Pletzer, Steve W. F. R. Waqanivavalagi, Paget Milsom, Nicolas P. Smith

**Affiliations:** ^1^Auckland Bioengineering Institute, The University of Auckland, Auckland, New Zealand; ^2^Green Lane Cardiothoracic Surgical Unit, Auckland City Hospital, Auckland, New Zealand; ^3^New Zealand eScience Infrastructure, Wellington, New Zealand; ^4^School of Mechanical, Medical and Process Engineering, Queensland University of Technology, Brisbane, QLD, Australia

**Keywords:** coronary artery bypass and grafting, total arterial grafting, graft configuration, computational fluid dynamics modeling, instantaneous wave-free ratio (iFR), transit-time flowmetry (TTFM), surgeon decision-making, surgical planning

## Abstract

**Objectives:**

Flow competition between coronary artery bypass grafts (CABG) and native coronary arteries is a significant problem affecting arterial graft patency. The objectives of this study were to compare the predictive hemodynamic flow resulting from various total arterial grafting configurations and to evaluate whether the use of computational fluid dynamics (CFD) models capable of predicting flow can assist surgeons to make better decisions for individual patients by avoiding poorly functioning grafts.

**Methods:**

Sixteen cardiac surgeons declared their preferred CABG configuration using bilateral internal mammary and radial arteries for each of 5 patients who had differing degrees of severe triple vessel coronary disease. Surgeons selected both a preferred 'aortic' strategy, with at least one graft arising from the ascending aorta, and a preferred “anaortic” strategy which could be performed as a “no-aortic touch” operation. CT coronary angiograms of the 5 patients were coupled to CFD models using a novel flow solver “COMCAB.” Twelve different CABG configurations were compared for each patient of which 4 were “aortic” and 8 were “anaortic.” Surgeons then selected their preferred grafting configurations after being shown predictive hemodynamic metrics including functional assessment of stenoses (instantaneous wave-free ratio; fractional flow reserve), transit time flowmetry graft parameters (mean graft flow; pulsatility index) and myocardial perfusion.

**Results:**

A total of 87.5% (7/8) of “anaortic” configurations compared to 25% (1/4) of “aortic” configurations led to unsatisfactory grafts in at least 1 of the 5 patients (*P* = 0.038). The use of the computational models led to a significant decrease in the selection of unsatisfactory grafting configurations when surgeons employed “anaortic” (21.25% (17/80) vs. 1.25% (1/80), *P* < 0.001) but not “aortic” techniques (5% (4/80) vs. 0% (0/80), *P* = 0.64). Similarly, there was an increase in the selection of ideal configurations for “anaortic” (6.25% (5/80) vs. 28.75% (23/80), *P* < 0.001) but not “aortic” techniques (65% (52/80) vs. 61.25% (49/80), *P* = 0.74). Furthermore, surgeons who planned to use more than one unique “anaortic” configuration across all 5 patients increased (12.5% (2/16) vs. 87.5% (14/16), *P*<0.001).

**Conclusions:**

“COMCAB” is a promising tool to improve personalized surgical planning particularly for CABG configurations involving composite or sequential grafts which are used more frequently in anaortic operations.

## 1. Introduction

Total arterial grafting (TAG), which involves the exclusive use of arterial conduits such as bilateral internal mammary arteries (BIMA) and radial arteries (RA) for coronary artery bypass grafting (CABG), has been associated with improved long-term clinical outcomes (1). Total arterial, anaortic, off-pump coronary artery bypass grafting (OPCABG) is an operative approach that avoids aortic manipulation with no graft attached to the ascending aorta and thus this strategy has been endorsed for its reduction in stroke risk and other complications associated with cardiopulmonary bypass (2). However, anaortic grafting configurations typically require more composite and sequential grafts rather than separate grafts.

Composite BIMA grafting to left coronary targets with <70% diameter stenosis leads to a high rate of graft occlusion or constriction due to competitive flow (3). For a radial artery graft anastomosed to the right coronary artery (RCA) territory, the percent diameter stenosis should be at least 80–90% because the radial artery is more prone to spasm (4, 5). Functional assessment of coronary stenoses using fractional flow reserve (FFR) < 0.80 or instantaneous wave-free ratio (iFR) < 0.90 (6), and functional assessment of bypass grafts using transit-time flowmetry (TTFM) with mean graft flow (MGF) ≥ 15 ml/min and pulsatility index (PI) < 5 can avoid situations of competitive flow leading to poor graft patency following arterial grafting (7).

There is no consensus among surgeons regarding the optimal TAG configuration using BIMA for an individual patient and it has even been argued that configuration is not important (8). However, others have maintained that graft configuration is significant (9) and that suboptimal judgement in the arrangement of grafts can lead to steal of flow between grafts and native coronary arteries particularly when composite or sequential grafts are used with unbalanced native coronary stenoses (10). In this study, the importance of graft configuration was investigated using predictive patient-specific hemodynamic computational modeling and the impact of such predictive information on surgical planning was evaluated.

## 2. Materials and methods

### 2.1. Selection of patient cases

**Figure 1:**
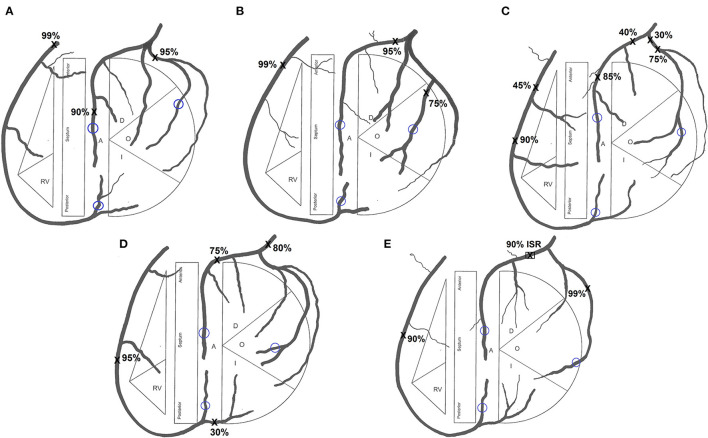
Coronary tree diagram for **(A)** Patient 1, **(B)** Patient 2, **(C)** Patient 3, **(D)** Patient 4 and **(E)** Patient 5. Sites of stenoses marked X. Sites of grafting encircled blue. *RV*, right ventricle; *A*, anterior; *O*, obtuse marginal; *I*, intermediate; *ISR*, in-stent restenosis.

Institutional ethics review was obtained to conduct this study. Five patient cases for inclusion in the study were identified by screening patients who had undergone both a CT coronary angiogram (CTCA) and invasive coronary angiogram. All patients were required to have severe triple-vessel coronary artery disease with >90% diameter stenosis in the RCA territory and >75% stenosis in the left anterior descending (LAD) and circumflex (CIRC) territories such that surgeons would not deliberately avoid using a RA graft on a distal target due to concerns of competitive flow. Patients 1 and 2 both had a ramus intermedius artery (Figures 1A, B), Patients 3 and 4 had additional less severe stenoses (Figures 1C, D), and Patient 5 had an in-stent restenosis in the LAD (Figure 1E). The differing degrees of severity and distribution of stenoses evident from the coronary tree diagrams of the five patients are summarized in Table 1.

**Table 1:** Distribution of severe stenoses in the five patient cases.

	**Location and percentage diameter severe stenoses in each territory**		
	**LAD**_territory_	**CIRC**_territory_	**RCA**_territory_
Patient 1	Mid LAD−90%	Prox CIRC−95%	Ostial RCA−99%
Patient 2	Prox LAD−95%	Prox OM1−75%	Prox RCA−99%
Patient 3	Mid LAD−85%	Prox CIRC−75%	Mid RCA−90%
Patient 4	Mid LMCA−80% Prox LAD−75%	Mid LMCA−80%	Mid RCA−95%
Patient 5	ISR Prox LAD−90%	Prox OM2−99%	Mid RCA−90%

Prox, stenosis in the proximal third of the vessel; Mid, stenosis in the middle third of the vessel; ISR, in-stent restenosis; LMCA, left main coronary artery; LAD, left anterior descending artery; CIRC, circumflex coronary artery; OM1, first obtuse marginal artery; OM2, second obtuse marginal artery; RCA, right coronary artery.

### 2.2. Selection of grafting configurations

**Figure 2:**
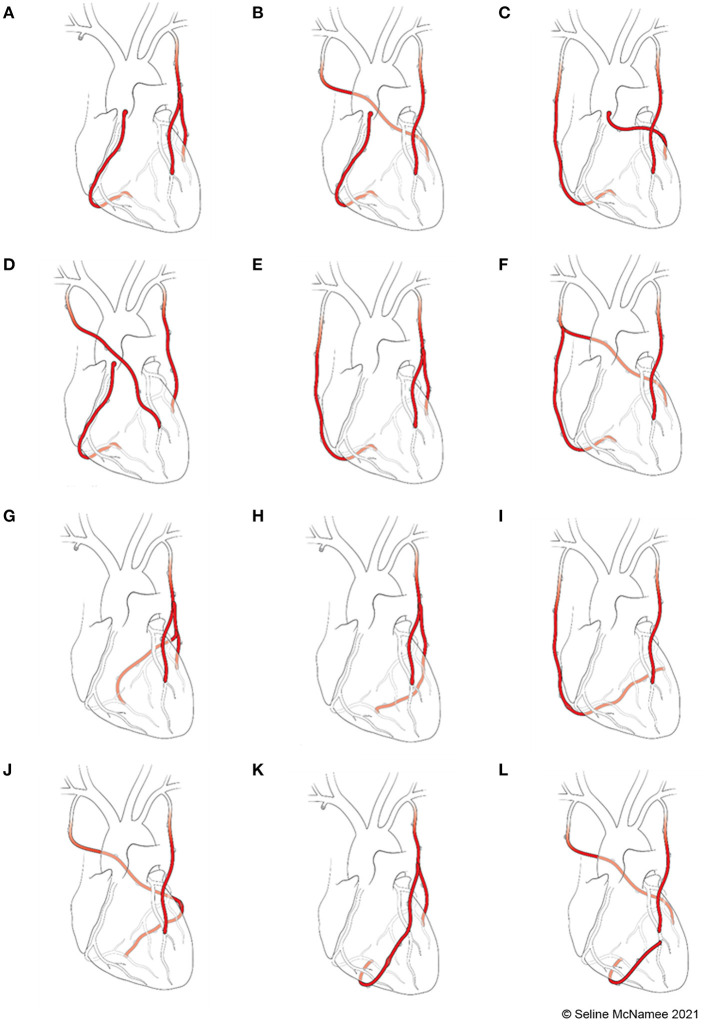
Total arterial grafting configurations. **(A)** *Configuration A: in situ* LIMA to LAD; free RIMA Y (off LIMA) to OM; free RIMA (off aorta) to PDA. **(B)** *Configuration B: in situ* LIMA to LAD; *in situ* RIMA (*via* transverse sinus) to OM; RA (off aorta) to PDA. **(C)** *Configuration C*: *in situ* LIMA to LAD; RA (off aorta) to OM; *in situ* RIMA to PDA. **(D)** *Configuration D: in situ* RIMA to LAD; *in situ* LIMA to OM; RA (off aorta) to PDA. **(E)** *Configuration E: in situ* LIMA to LAD; RA Y (off LIMA) to OM; *in situ* RIMA to PDA. **(F)** *Configuration F: in situ* LIMA to LAD; *in situ* RIMA to OM; RA Y (off RIMA) to PDA. **(G)** *Configuration G: in situ* LIMA to LAD; free RIMA Y (off LIMA) to OM; RA Y (off RIMA) to PDA. **(H)** *Configuration H: in situ* LIMA to LAD; free RIMA Y (off LIMA) to OM sequential to PDA. **(I)** *Configuration I: in situ* LIMA to LAD; *in situ* RIMA to PDA with RA I (extends RIMA) sequential to OM. **(J)** *Configuration J: in situ* LIMA to LAD; *in situ* RIMA to OM with RA I (extends RIMA) sequential to PDA. **(K)** *Configuration K: in situ* LIMA to OM; free RIMA Y (off LIMA) to LAD with RA I (extends RIMA) sequential to PDA. **(L)** *Configuration L: in situ* LIMA to LAD; *in situ* RIMA to OM; RA J (off LAD) to PDA.

Twelve grafting configurations utilizing BIMA and RA, used by surgeons internationally, were examined for each of the 5 patient-specific diseased coronary circulations. Four of the grafting configurations involved aorto-coronary grafts (“aortic” configurations). One configuration using BIMA exclusively, recycles the free RIMA for use to the RCA by anastomosing it from the aorta while using a LIMA/RIMA composite graft for the left-sided vessels (11) *(configuration A)* (Figure 2A). Other configurations have three separate inflows with separate *in situ* use of both internal mammary arteries (IMA) and the RA off the aorta. The RIMA can be used *in situ via* the transverse sinus to the CIRC branches (12) *(configuration B)* (Figure 2B), *in situ* to the distal RCA or proximal posterior descending artery (PDA) (13) *(configuration C)* (Figure 2C), or, if used for the LAD, then the LIMA is anastomosed to the CIRC branches (14) *(configuration D)* (Figure 2D).

The other eight grafting configurations involved no proximal graft anastomosis to the aorta (“anaortic” configurations). A composite Y-graft can be constructed using the RA off the *in situ* LIMA to LAD and if the *in situ* RIMA cannot reach the RCA target then it is lengthened by a small I-graft with the remaining RA (15) *(configuration E)* (Figure 2E). An alternative configuration involves the RIMA in the Y-composite graft which spares the LIMA to be used as an individual graft (16) *(configuration F)* (Figure 2F). Certain surgeons perform a double-Y graft to revascularize all three territories and this is based on a single inflow (17) *(configuration G)* (Figure 2G). Another composite approach used by surgeons, based on a single inflow requiring use of the BIMA only, is a configuration that uses the free RIMA as a sequential graft (11, 18) *(configuration H)* (Figure 2H).

The appeal in using both IMA as *in situ* grafts, has led surgeons to using the RA as an I-graft to lengthen the RIMA and configure it as a sequential graft to anastomose the CIRC and RCA territories. The *in situ* RIMA-RA sequential can be used in an anticlockwise orientation where the first anastomosis is to the RCA and the final anastomosis is to the CIRC branches (19) *(configuration I)* (Figure 2I). The orientation of the RIMA-RA sequential in the clockwise version is such that it is first brought through the transverse sinus to anastomose to the CIRC branches and then terminates on the RCA (20) *(configuration J)* (Figure 2J). Another configuration uses an I-graft constructed with the RIMA and RA but uses it as a free Y- graft from the LIMA to CIRC (21) *(configuration K)* (Figure 2K). Finally, an uncommon “bail-out” configuration involves the use of a jump graft where the radial artery conduit is anastomosed from the distal LAD to the PDA (22) *(configuration L)* (Figure 2L).

### 2.3. Computational model analysis of grafting configurations

Patient-specific 1D-0D computational fluid dynamics models were created for each of the 5 patients' theoretical non-diseased coronary circulation, their stenotic circulation and their 12 grafted circulations and these 70 networks were solved using the novel software “COMCAB” created by the authors for this purpose. This involved a manual segmentation of the native coronary artery geometry from the CTCA for each patient and the mapping of each vessel onto the 1D domain. Thereafter, coronary artery side branches were added to account for a physiological loss in pressure from proximal to distal along the coronary arteries as such branches were not visualized on the CTCA. Terminal vessels were coupled to 0D lumped parameter models. Lumped parameter stenoses were added to create the stenotic network for each patient and 1D grafts added to create the different grafting network topologies for the grafted circulations. The networks were solved for blood flow and pressure using the Richtmyer two-step Lax-Wendroff 1D numerical method (23, 24) with prescription of appropriate boundary conditions. The chosen meshgrid size, Δ*x*, was set at Δ*x* ≈ 0.1cm and the timestep, Δ*t*, was chosen to be the largest value possible whilst ensuring stability of the numerical scheme with Δ*t* ≈ 2.95 × 10^−5^. The methodology for the creation of the CFD models has previously been described in detail in the literature (25). The vessel segment data and vessel segment relations used for all network simulations for the five patients are provided in Supplementary material 1–5. The computational model inputs included a generic aortic root pressure waveform of 120/77 mmHg at 65 beats per minute (cardiac period 0.917 s), a generic left ventricular (LV) pressure waveform, and cardiac output (CO) of 5 L/min. Approximately 4.5% of CO was assigned to myocardial blood flow with this flow distributed to the three main coronary territories: LAD, CIRC, and RCA, as dictated by distribution of vessels on the CTCA. Total arterial compliance was estimated at 1.15 cm^3^/mmHg from the aortic pressure waveform and a 3-element Windkessel resistance-capacitance-resistance (RCR) model was applied at the outlets of the terminal vessels to represent the distal microcirculation (25). iFR was chosen as the metric for functional stenosis severity as it is measured at rest like TTFM graft measurements, not at hyperemia which is used for FFR. However, as iFR is a recent concept an equivalent FFR was also calculated: *FFR* = 0.68 × *iFR* + 0.18 (26).

**Figure 3:**
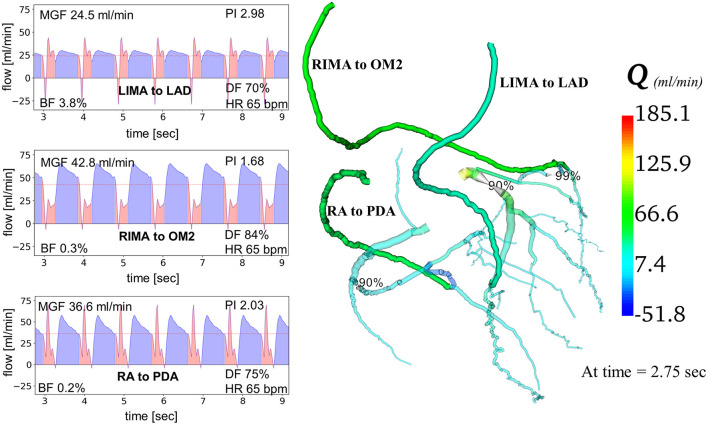
Computational modeling of coronary artery bypass grafts using “COMCAB” software. An example is shown of how the computational model displays predictive graft performance data measured at the distal end of the grafts from grafting configuration B for Patient 5. *Q*, flow; *LIMA*, left internal mammary artery; *RIMA*, right internal mammary artery; *RA*, radial artery; *LAD*, left anterior descending artery; *OM2*, second obtuse marginal artery; *PDA*, posterior descending artery; *MGF*, mean graft flow; *PI*, pulsatility index; *BF*, backward flow; *DF*, diastolic filling; *HR*, heart rate.

Creation of the predictive models took ~6 h for each patient. Running all simulations using high performance computing on Xeon Broadwell CPUs (2.1 GHz) with embarrassing parallelisation, took up to 75 min. MGF and PI were calculated at the distal end of each graft (Figure 3). Myocardial territory perfusion was calculated for the theoretical non-diseased circulations and the diseased circulations along with the improvement in myocardial perfusion following restoration of blood flow by the grafted coronary circulations. Validation of the computational models' predictions was performed by comparing the calculated iFR, MGF and PI with *in vivo* measurements available in the literature.

Grafting configurations were classified as unsatisfactory, satisfactory, or ideal, based on the graft performance indices. An unsatisfactory grafting configuration was defined as having either MGF < 15 ml/min or PI > 5 in any graft. A satisfactory grafting configuration was defined as MGF ≥ 15 ml/min in all grafts with 3 < PI < 5 for a graft to a LV target and an ideal grafting configuration was defined as MGF ≥ 15 ml/min and PI < 3 in all grafts (27).

### 2.4. Selection of participant surgeons

Sixteen cardiac surgeons were recruited for participation in the study from five centers. The power calculation assumed that ~50% of the selected grafting configurations would be deemed unsatisfactory with standard surgeon decision-making but close to 0% after the provision of computer modeling predictions. Thus, the study would require a sample size of 16 to achieve two-tailed statistical significance with an alpha level of 0.05 and a power of 0.8. No dropouts were expected due to the administration of the survey in one sitting. Surgeon experience was noted by recording the volume of CABG operations performed in their career and current practice of total arterial revascularisation using BIMA.

### 2.5. Survey administration procedures

The coronary tree diagrams, as representations of each patient's diseased coronary circulation, were presented to the surgeons (Figure 1). Surgeons were informed that the only available conduits would be the LIMA, RIMA and one RA with no contraindications for their use. All patients had an overall preserved LV function with all distal grafting target sites being suitable for grafting. Conduit lengths were adequate for an *in situ* RIMA to reach the obtuse marginal (OM) target and for an *in situ* RIMA to reach a distal RCA/PDA.

Each of the surgeons selected one preferred “aortic” configuration and one preferred “anaortic” configuration for each of the five patients (standard decision-making). They were then asked to indicate their overall grafting preference if there were no constraints on their choice such as a porcelain aorta. The surgeons were shown the 12 grafting configurations used by surgeons internationally and asked to rank their top five selections as well as indicate any configurations they would not use. They were then presented with the predictive hemodynamic graft flow information from the computer model and asked for their preferred selections before, and after, the provision of regional myocardial perfusion data (computer-informed decision-making) (Tables 2–6).

Table 2Hemodynamic predictions for Patient 1.

**Table:** 

**Stenosis location**	**Stenosis length (cm)**	**% Diameter stenosis**	**iFR**	**FFR**	**Region**	**Regional perfusion (ml/min)**	**No disease (ml/min)**
**Functional significance of stenoses**							
LAD	2.2	90	0.73	0.68	LAD	99.17	106.48
CIRC	0.7	95	0.50	0.52	CIRC	25.43	75.76
RCA	0.6	99	0.29	0.38	RCA	28.68	91.74
					*Total*	*153.28*	*273.98*

**Table:** 

**Aortic configuration**	**Region**	**Graft**	**MGF (ml/min)**	**PI**	**Regional perfusion (ml/min)**
**Functional graft performance**					
A	LAD	LIMA to LAD	17.57	1.54	105.92
	CIRC	RIMA to OM1	45.01	1.61	61.88
	RCA	RIMA to PDA	61.91	1.24	84.56
					*252.36*
B	LAD	LIMA to LAD	21.47	1.30	107.39
	CIRC	RIMA to OM1	44.99	1.52	61.84
	RCA	Radial to PDA	64.99	1.46	87.33
					*256.57*
C	LAD	LIMA to LAD	21.47	1.30	107.40
	CIRC	Radial to OM1	56.08	1.24	70.75
	RCA	RIMA to PDA	55.01	1.20	78.40
					*256.55*
D	LAD	RIMA to LAD	20.59	1.43	107.07
	CIRC	LIMA to OM1	45.86	1.56	62.56
	RCA	Radial to PDA	64.99	1.46	87.33
					*256.96*
E	LAD	LIMA to LAD	17.20	1.65	105.77
	CIRC	Radial to OM1	49.39	1.61	65.37
	RCA	RIMA to PDA	55.01	1.20	78.40
					*249.55*
F	LAD	LIMA to LAD	21.47	1.31	107.39
	CIRC	RIMA to OM1	40.09	1.65	57.91
	RCA	Radial to PDA	58.47	1.43	81.50
					*246.81*
G	LAD	LIMA to LAD	13.75	1.65	104.47
	CIRC	RIMA to OM1	37.75	1.84	56.04
	RCA	Radial to PDA	51.68	1.44	75.32
					*235.83*
H	LAD	LIMA to LAD	14.66	1.61	104.81
	CIRC	RIMA to OM1	78.94	0.91	54.91
	RCA	RIMA to PDA	42.65	1.15	67.23
					*226.95*
I	LAD	LIMA to LAD	21.47	1.31	107.39
	CIRC	Radial to OM1	29.56	2.28	49.46
		RCA	RIMA to PDA	77.33	1.05	71.88
					*228.73*
J	LAD	LIMA to LAD	21.47	1.31	107.39
	CIRC	RIMA to OM1	77.04	0.96	51.70
	RCA	Radial to PDA	44.74	1.10	69.11
					*228.20*
K	LAD	RIMA to LAD	54.75	1.95	102.49
	CIRC	LIMA to OM1	40.91	1.75	58.57
	RCA	Radial to PDA	46.23	1.48	70.48
					*231.54*
L	LAD	LIMA to LAD	51.13	1.10	102.43
	CIRC	RIMA to OM1	44.99	1.52	61.84
	RCA	Radial to PDA	38.51	1.05	63.50
					*227.77*

Values in red indicate PI > 5 or mean graft flow < 15 ml/min; blue indicate PI > 3 for graft to left-sided target. MGF, mean graft flow; PI, pulsatility index; LIMA, left internal mammary artery; RIMA, right internal mammary artery; RA, radial artery; LAD, left anterior descending coronary artery; CIRC, circumflex coronary artery; OM1, first obtuse marginal artery; RCA, right coronary artery; PDA, posterior descending coronary artery. Italicized values indicate total myocardial perfusion.

Table 3Hemodynamic predictions for Patient 2.

**Table:** 

**Stenosis location**	**Stenosis length (cm)**	**% Diameter stenosis**	**iFR**	**FFR**	**Region**	**Regional perfusion (ml/min)**	**No disease (ml/min)**
**Functional significance of stenoses**							
LAD	0.9	95	0.85	0.76	LAD	101.12	116.91
OM1	2	75	0.86	0.77	CIRC	56.76	60.97
RCA	1.8	99	0.23	0.34	RCA	21.05	74.36
					*Total*	*178.92*	*252.24*

**Table:** 

**Aortic configuration**	**Region**	**Graft**	**MGF (ml/min)**	**PI**	**Regional perfusion (ml/min)**
**Functional graft performance**					
	LAD	LIMA to LAD	37.92	1.33	115.87
A	CIRC	RIMA to OM1	14.20	4.90	60.18
	RCA	RIMA to PDA	49.37	0.76	67.90
					*243.96*
	LAD	LIMA to LAD	40.61	1.48	116.92
B	CIRC	RIMA to OM1	19.57	3.58	61.39
	RCA	Radial to PDA	51.12	0.85	69.57
					*247.88*
	LAD	LIMA to LAD	40.57	1.49	116.95
C	CIRC	Radial to OM1	27.92	2.73	63.32
	RCA	RIMA to PDA	45.11	0.92	63.87
					*244.15*
	LAD	RIMA to LAD	36.81	1.61	115.50
D	CIRC	LIMA to OM1	20.02	3.68	61.51
	RCA	Radial to PDA	51.12	0.85	69.57
					*246.58*
	LAD	LIMA to LAD	37.45	1.34	115.70
E	CIRC	Radial to OM1	16.54	4.92	60.69
	RCA	RIMA to PDA	45.11	0.92	63.87
					*240.26*
	LAD	LIMA to LAD	40.63	1.49	116.89
F	CIRC	RIMA to OM1	13.67	5.20	60.05
	RCA	Radial to PDA	48.41	0.94	67.01
					*243.95*
	LAD	LIMA to LAD	31.14	1.64	113.26
G	CIRC	RIMA to OM1	4.52	16.44	57.93
	RCA	Radial to PDA	44.38	0.84	63.17
					*234.36*
	LAD	LIMA to LAD	32.68	1.56	113.83
H	CIRC	RIMA to OM1	41.15	1.47	57.25
	RCA	RIMA to PDA	39.62	0.68	58.66
					*229.74*
	LAD	LIMA to LAD	40.68	1.48	116.83
**I**	CIRC	Radial to OM1	0.68	114.35	57.06
	RCA	RIMA to PDA	45.60	1.21	63.71
					*237.61*
	LAD	LIMA to LAD	40.68	1.48	116.84
J	CIRC	RIMA to OM1	44.59	1.31	57.27
	RCA	Radial to PDA	43.04	0.63	61.91
					*236.02*
	LAD	RIMA to LAD	58.93	1.41	109.01
K	CIRC	LIMA to OM1	10.71	6.86	59.32
	RCA	Radial to PDA	39.02	0.93	58.10
					*226.43*
	LAD	LIMA to LAD	54.69	1.05	109.82
L	CIRC	RIMA to OM1	19.59	3.57	61.37
	RCA	Radial to PDA	27.02	0.87	46.70
					*217.90*

Values in red indicate PI > 5 or mean graft flow < 15 ml/min; blue indicate PI > 3 for graft to left-sided target. MGF, mean graft flow; PI, pulsatility index; LIMA, left internal mammary artery; RIMA, right internal mammary artery; RA, radial artery; LAD, left anterior descending coronary artery; CIRC, circumflex coronary artery; OM1, first obtuse marginal artery; RCA, right coronary artery; PDA, posterior descending coronary artery.

Table 4Hemodynamic predictions for Patient 3.

**Table:** 

**Stenosis location**	**Stenosis length (cm)**	**% Diameter stenosis**	**iFR**	**FFR**	**Region**	**Regional perfusion (ml/min)**	**No disease (ml/min)**
**Functional significance of stenoses**							
LAD1	0.5	40	0.99	0.85	LAD	80.83	90.49
LAD2	1.1	85	0.84	0.75	CIRC	69.64	83.57
CIRC1	0.4	30	1.00	0.86	RCA	55.52	70.50
CIRC2	0.8	75	0.82	0.74	*Total*	*205.99*	*244.56*
RCA1	0.9	45	0.98	0.85			
RCA2	2	90	0.53	0.54

**Table:** 

**Aortic configuration**	**Region**	**Graft**	**MGF (ml/min)**	**PI**	**Regional perfusion (ml/min)**
**Functional graft performance**					
	LAD	LIMA to LAD	26.73	1.41	91.18
A	CIRC	RIMA to OM1	34.10	2.05	85.51
	RCA	RIMA to PDA	28.57	1.02	75.99
					*252.69*
	LAD	LIMA to LAD	31.25	1.43	92.88
B	CIRC	RIMA to OM1	36.19	1.89	86.48
	RCA	Radial to PDA	29.32	1.24	76.53
					*255.89*
	LAD	LIMA to LAD	31.18	1.43	92.95
C	CIRC	Radial to OM1	50.41	1.39	92.86
	RCA	RIMA to PDA	26.72	1.34	74.69
					*260.51*
	LAD	RIMA to LAD	29.24	1.53	92.15
D	CIRC	LIMA to OM1	37.17	1.93	86.93
	RCA	Radial to PDA	29.32	1.24	76.53
					*255.60*
	LAD	LIMA to LAD	26.03	1.48	90.95
E	CIRC	Radial to OM1	39.23	2.05	87.80
	RCA	RIMA to PDA	26.72	1.34	74.69
					*253.44*
	LAD	LIMA to LAD	31.26	1.43	92.86
F	CIRC	RIMA to OM1	32.99	2.11	85.04
	RCA	Radial to PDA	27.05	1.35	74.92
					*252.82*
	LAD	LIMA to LAD	24.17	1.56	90.20
G	CIRC	RIMA to OM1	29.76	2.44	83.54
	RCA	Radial to PDA	23.94	1.40	72.71
					*246.44*
	LAD	LIMA to LAD	24.75	1.52	90.40
H	CIRC	RIMA to OM1	49.44	1.29	82.86
	RCA	RIMA to PDA	21.22	1.32	70.78
					*244.04*
	LAD	LIMA to LAD	31.30	1.43	92.82
I	CIRC	Radial to OM1	24.68	2.89	81.28
	RCA	RIMA to PDA	47.96	1.11	72.28
					*246.38*
	LAD	LIMA to LAD	31.25	1.43	92.88
J	CIRC	RIMA to OM1	36.19	1.90	86.48
	RCA	Radial to PDA	29.87	1.03	76.93
					*256.29*
	LAD	RIMA to LAD	41.47	2.06	88.56
K	CIRC	LIMA to OM1	31.87	2.26	84.45
	RCA	Radial to PDA	21.78	1.68	71.18
					*244.19*
	LAD	LIMA to LAD	36.45	1.23	89.35
**L**	CIRC	RIMA to OM1	36.20	1.89	86.47
	RCA	Radial to PDA	9.92	3.03	62.69
					*238.52*

Values in red indicate PI > 5 or mean graft flow < 15 ml/min; blue indicate PI > 3 for graft to left-sided target. MGF, mean graft flow; PI, pulsatility index; LIMA, left internal mammary artery; RIMA, right internal mammary artery; RA, radial artery; LAD, left anterior descending coronary artery; CIRC, circumflex coronary artery; OM1, first obtuse marginal artery; RCA, right coronary artery; PDA, posterior descending coronary artery.

Table 5Hemodynamic predictions for Patient 4.

**Table:** 

**Stenosis location**	**Stenosis length (cm)**	**% Diameter stenosis**	**iFR**	**FFR**	**Region**	**Regional perfusion (ml/min)**	**No disease (ml/min)**
**Functional significance of stenoses**							
LMCA	1.2	80	0.89	0.79	LAD	79.88	94.54
LAD	0.8	75	0.94	0.82	CIRC	61.31	71.72
RCA2	1.5	95	0.56	0.56	RCA	51.84	81.55
PLB	1	30	0.95	0.83	*Total*	*193.04*	*247.81*

**Table:** 

**Aortic configuration**	**Region**	**Graft**	**MGF (ml/min)**	**PI**	**Regional perfusion (ml/min)**
**Functional graft performance**					
	LAD	LIMA to LAD	38.82	0.99	98.88
A	CIRC	RIMA to OM2	30.07	2.75	74.26
	RCA	RIMA to PDA	46.88	0.83	78.67
					*251.81*
	LAD	LIMA to LAD	42.99	1.07	100.95
B	CIRC	RIMA to OM2	34.49	2.32	76.03
	RCA	Radial to PDA	49.71	1.04	80.24
					*257.23*
	LAD	LIMA to LAD	42.51	1.08	101.54
C	CIRC	Radial to OM2	49.54	1.77	81.47
	RCA	RIMA to PDA	40.79	1.26	75.23
					*258.24*
	LAD	RIMA to LAD	39.95	1.15	99.69
D	CIRC	LIMA to OM2	35.61	2.37	76.31
	RCA	Radial to PDA	49.71	1.04	80.24
					*256.24*
	LAD	LIMA to LAD	37.93	1.03	98.75
E	CIRC	Radial to OM2	35.10	2.78	76.04
	RCA	RIMA to PDA	40.79	1.26	75.23
					*250.02*
	LAD	LIMA to LAD	43.17	1.07	100.73
F	CIRC	RIMA to OM2	28.88	2.78	74.02
	RCA	Radial to PDA	43.91	1.31	76.99
					*251.74*
	LAD	LIMA to LAD	35.07	1.07	96.82
G	CIRC	RIMA to OM2	22.40	3.87	71.32
	RCA	Radial to PDA	35.80	1.28	72.42
					*240.55*
	LAD	LIMA to LAD	36.31	1.04	97.28
H	CIRC	RIMA to OM2	49.84	1.46	70.84
	RCA	RIMA to PDA	28.95	1.04	68.53
					*236.66*
	LAD	LIMA to LAD	43.54	1.06	100.27
I	CIRC	Radial to OM2	17.34	4.94	69.87
	RCA	RIMA to PDA	53.14	1.12	72.43
					*242.58*
	LAD	LIMA to LAD	43.45	1.06	100.39
J	CIRC	RIMA to OM2	53.22	1.23	70.93
	RCA	Radial to PDA	33.00	0.99	70.83
					*242.15*
	LAD	RIMA to LAD	57.17	1.55	94.37
K	CIRC	LIMA to OM2	27.34	3.14	72.82
	RCA	Radial to PDA	28.33	1.69	68.18
					*235.37*
	LAD	LIMA to LAD	48.58	1.03	97.29
L	CIRC	RIMA to OM2	34.58	2.32	75.96
	RCA	Radial to PDA	10.53	2.61	57.97
					*231.22*

Values in red indicate PI > 5 or mean graft flow < 15 ml/min; blue indicate PI > 3 for graft to left-sided target. MGF, mean graft flow; PI, pulsatility index; LIMA, left internal mammary artery; RIMA, right internal mammary artery; RA, radial artery; LAD, left anterior descending coronary artery; CIRC, circumflex coronary artery; OM2, second obtuse marginal artery; RCA, right coronary artery; PDA, posterior descending coronary artery.

Table 6Hemodynamic predictions for Patient 5.

**Table:** 

**Stenosis location**	**Stenosis length (cm)**	**% Diameter stenosis**	**iFR**	**FFR**	**Region**	**Regional perfusion (ml/min)**	**No disease (ml/min)**
**Functional significance of stenoses**							
LAD	1.6	90	0.93	0.81	LAD	87.48	95.95
OM2	1.2	99	0.29	0.38	CIRC	33.27	61.69
RCA	1.5	90	0.73	0.68	RCA	57.83	71.40
						*178.59*	*229.04*

**Table:** 

**Aortic configuration**	**Region**	**Graft**	**MGF (ml/min)**	**PI**	**Regional perfusion (ml/min)**
**Functional graft performance**					
	LAD	LIMA to LAD	10.77	7.11	88.99
A	CIRC	RIMA to OM2	43.34	1.67	76.51
	RCA	RIMA to PDA	34.16	1.82	70.98
					*236.48*
	LAD	LIMA to LAD	24.47	2.98	90.80
B	CIRC	RIMA to OM2	42.79	1.68	75.95
	RCA	Radial to PDA	36.60	2.03	71.88
					*238.63*
	LAD	LIMA to LAD	24.47	2.98	90.80
C	CIRC	Radial to OM2	50.40	1.50	83.53
	RCA	RIMA to PDA	29.02	2.07	69.07
					*243.40*
	LAD	RIMA to LAD	21.09	3.21	90.37
D	CIRC	LIMA to OM2	43.41	1.74	76.60
	RCA	Radial to PDA	36.60	2.03	71.88
					*238.84*
	LAD	LIMA to LAD	9.78	8.40	88.85
E	CIRC	Radial to OM2	46.51	1.69	79.64
	RCA	RIMA to PDA	29.02	2.08	69.06
					*237.55*
	LAD	LIMA to LAD	24.47	2.98	90.80
F	CIRC	RIMA to OM2	40.95	1.75	74.10
	RCA	Radial to PDA	29.37	2.46	69.18
					*234.08*
	LAD	LIMA to LAD	4.11	18.48	88.10
G	CIRC	RIMA to OM2	41.81	1.76	74.95
	RCA	Radial to PDA	22.77	2.65	66.68
					*229.73*
	LAD	LIMA to LAD	6.31	11.33	88.40
H	CIRC	RIMA to OM2	57.54	1.21	74.82
	RCA	RIMA to PDA	15.87	2.59	64.04
					*227.26*
	LAD	LIMA to LAD	24.47	2.98	90.80
I	CIRC	Radial to OM2	37.41	1.97	70.56
	RCA	RIMA to PDA	54.42	1.34	64.49
					*225.85*
	LAD	LIMA to LAD	24.47	2.98	90.80
J	CIRC	RIMA to OM2	54.85	1.16	72.72
	RCA	Radial to PDA	15.29	3.04	63.81
					*227.33*
	LAD	RIMA to LAD	18.02	4.17	86.77
K	CIRC	LIMA to OM2	42.85	1.72	75.99
	RCA	Radial to PDA	23.14	1.97	66.84
					*229.60*
	LAD	LIMA to LAD	23.83	3.05	92.09
L	CIRC	RIMA to OM2	42.79	1.68	75.95
	RCA	Radial to PDA	−2.99	9.80	56.71
					*224.76*

Values in red indicate PI > 5 or mean graft flow < 15 ml/min; blue indicate PI > 3 for graft to left-sided target. MGF, mean graft flow; PI, pulsatility index; LIMA, left internal mammary artery; RIMA, right internal mammary artery; RA, radial artery; LAD, left anterior descending coronary artery; CIRC, circumflex coronary artery; OM2, second obtuse marginal artery; RCA, right coronary artery; PDA, posterior descending coronary artery.

### 2.6. Outcomes of interest

The outcomes of interest were the selected number of unsatisfactory graft configurations (primary outcome measure), the number of ideal graft configurations, and the number of unique grafting configurations among the five patients for both “aortic” and “anaortic” configurations. Additional exploratory measures included the number of configuration rankings that changed with additional regional myocardial perfusion data and the number of configurations that individual surgeons indicated they would not initially use but later would use after viewing the computational models' hemodynamic predictions.

### 2.7. Statistical analysis

Differences in outcome measures were compared as continuous variables for flows with paired *t*-tests and as proportions for discrete variables with chi-squared tests. If parametric assumptions were not satisfied, then the Mann–Whitney *U*-test for independent comparisons was performed or the Wilcoxon signed rank-test for dependent comparisons. For the primary outcome measure, McNemar's test was performed to determine the influence of the computational model in changing individual surgeons' selection of unsatisfactory grafting configurations. Outcomes were considered statistically significant for *P* < 0.05 and the statistical tests were two-tailed with power 0.8 to test hypotheses. A learning gain attributable the computational model was set at 30% of surgeons improving their unsatisfactory decisions, which is considered effective for an educational intervention (28).

## 3. Results

### 3.1. Validation of computational fluid dynamics model predictions

**Figure 4:**
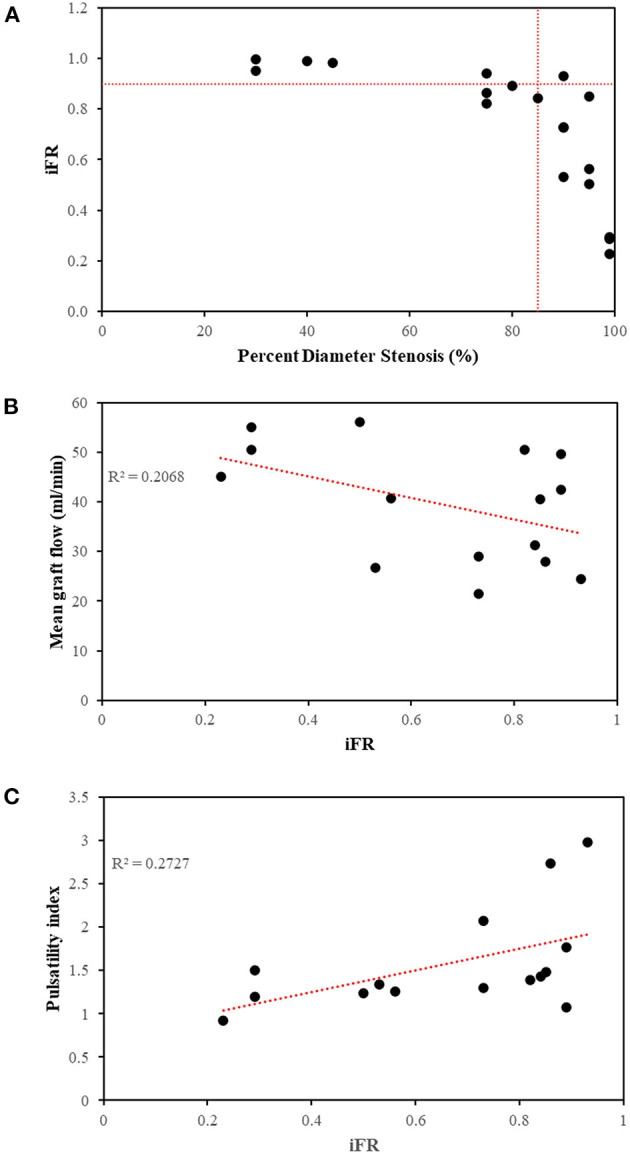
Relationships between metrics of functional stenoses and grafts. **(A)** The majority of stenoses >85% diameter were functionally significant at rest (iFR < 0.90). Two stenoses between 75 and 80% were also functionally significant on account of their increased lesion length. **(B)** Correlation between mean graft flow and iFR (*R* = −0.455) and **(C)** correlation between pulsatility index and iFR (*R* = 0.522) using the 15 arterial grafts across 5 patients using separate arterial grafts from configuration C, as an example. *iFR*, instantaneous wave-free ratio; *R*^2^, coefficient of determination.

Seven out of the 9 stenoses (78%) in this study that were between 71 and 90% diameter stenosis were functionally significant (Figure 4A). This proportion correlated with the findings of a large clinical study in which 80% of such lesions were found to be functionally significant (29). The MGF and PI predicted by the computational models compared well with available *in vivo* TTFM measurements from other clinical studies, noting that the measurement of PI depends on location along the graft and is graft-specific (Table 7). Furthermore, the correlation between MGF and iFR (*R* = −0.455) (Figure 4B) as well as PI and iFR (*R* = 0.522) for 15 separate arterial grafts in this study (Figure 4C) was consistent with an *in vivo* clinical study involving 25 arterial grafts where the correlation coefficients were *R* = −0.460 and 0.563, respectively (30).

**Table 7:** Validation of grafting predictions.

**Graft**	**“COMCAB” predictions**	***In vivo* clinical study**
***In situ*** **separate LIMA to LAD**	**Configurations B, C, F, I, J** **(*****n*** **=** **25)**	**Onorati et al**. **(****46****)** **(*****n*** **=** **69);** **Han et al**. **(****47****)** **(*****n*** **=** **20)**
MGF	32.48 ± 9.19	34.1 ± 21.4; 30.7 ± 10.3
PI	1.79 ± 0.71 (start) 1.66 ± 0.68 (end)	2.2 ± 0.5; 2.2 ± 0.6
***In situ*** **separate RIMA to LAD**	**Configuration D** **(*****n*** **=** **5)**	**Zhang et al**. **(****48****)** **(*****n*** **=** **34)**
MGF	29.53 ± 8.85	29.03 ± 22.73
PI	3.20 ± 1.10 (start) 1.78 ± 0.81 (end)	2.56 ± 0.96
***In situ*** **separate RIMA to CIRC**	**Configurations B, L** **(*****n*** **=** **10)**	**Han et al**. **(****47****)** **(*****n*** **=** **31)**
MGF	35.62 ± 9.41	33.4 ± 24.1
PI	2.49 ± 0.63 (start) 2.20 ± 0.78 (end)	2.3 ± 0.6
***In situ*** **separate RIMA to RCA/PDA**	**Configurations C, E** **(*****n*** **=** **10)**	**Han et al**. **(****47****)** **(*****n*** **=** **20)**
MGF	39.33 ± 11.02	51.7 ± 34.4
PI	3.08 ± 0.78 (start) 1.36 ± 0.41 (end)	2.1 ± 1.1
**Composite LIMA to LAD**	**Configurations A, E, G, H** **(*****n*** **=** **20)**	**Onorati et al**. **(****46****)** **(*****n*** **=** **42)**
MGF	24.16 ± 11.6	32.9 ± 25.6
PI	2.17 ± 2.76 (start) 3.39 ± 4.55 (end)	2.1 ± 0.4
**free RIMA Y off LIMA or aorta to LAD or CIRC**	**Configuration A** **(*****n*** **=** **5)**	**Han et al**. **(****47****)** **(*****n*** **=** **23)**
MGF	33.34 ± 12.39	28.9 ± 17.2
PI	1.56 ± 0.53 (start) 2.60 ± 1.37 (end)	3.2 ± 3.7
**RA Y off LIMA to RCA or CIRC**	**Configuration E** **(*****n*** **=** **5)**	**Onorati et al**. **(****46****)** **(*****n*** **=** **42)**
MGF	37.35 ± 12.95	36.5 ± 9.5
PI	1.64 ± 0.49 (start) 2.61 ± 1.37 (end)	1.8 ± 0.5
**RA off aorta to RCA or CIRC**	**Configurations B, C, D** **(*****n*** **=** **15)**	**Onorati et al**. **(****46****)** **(*****n*** **=** **69)**
MGF	46.52 ± 11.98^*^	35.9 ± 10.9
PI	4.07 ± 1.24 (start)^++^ 1.46 ± 0.51 (end)	2.3 ± 1.0

* RCA lesions were > 90% in this study whereas Onorati et al. (46) had >80%.

++ Affected by head of pressure from aorta (25).

Values are presented as mean ± standard deviation. MGF, mean graft flow; PI, pulsatility index; LIMA, left internal mammary artery; RIMA, right internal mammary artery; RA, radial artery; LAD, left anterior descending coronary artery; CIRC, circumflex coronary artery; RCA, right coronary artery; PDA, posterior descending coronary artery.

### 3.2. Patient-specific grafting configuration predictions

For Patient 1, configurations G and H had MGF < 15 ml/min in the composite LIMA to LAD graft mainly due to lower flows down this limb where the LIMA was the single inflow source for grafts to all three targets (Table 2). For Patient 2, configurations A, F, G, I and K had MGF < 15 ml/min and higher PI because composite grafts to the OM1 were subject to steal of flow, which was exacerbated by flow competition from a native OM1 75% stenosis with iFR 0.86 (Table 3). For Patients 3 and 4 only configuration L was unsatisfactory due to 90% and 95% stenosis, respectively, in the RCA that compromised the MGF and PI in the RA to PDA jump grafts (Tables 4, 5). For Patient 5, configurations A, E, G, and H were unsatisfactory with MGF < 15 ml/min in the LIMA to LAD graft due to a steal of blood flow down the other limb of the composite Y-graft, which was accentuated by the native competitive flow in the LAD due to a stenosis of 90% with an iFR 0.93 (Table 6). For Patient 5, configuration L was unsatisfactory due to a reversal of flow in the RA to PDA jump graft from a 90% RCA stenosis combined with limited blood flow supplied by the upstream LIMA to LAD (Table 6).

### 3.3. Comparison of “aortic” and “anaortic” grafting predictions

**Figure 5:**
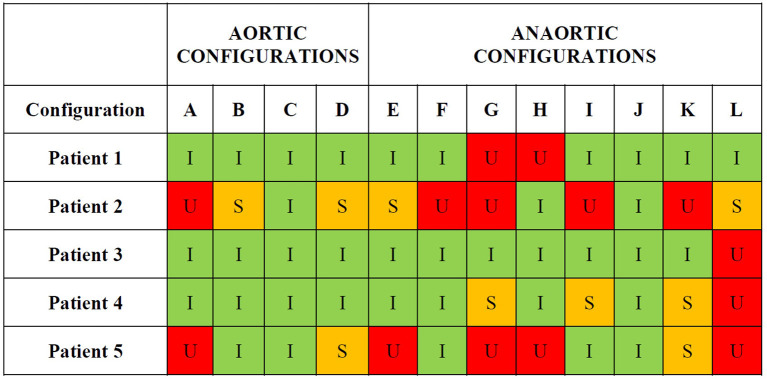
Unsatisfactory, satisfactory and ideal patient-specific grafting configurations. Out of 60 grafting configurations studied amongst the 5 patients, 14 were deemed unsatisfactory, 9 satisfactory and 37 ideal based on MGF and PI of individual grafts. *U*, unsatisfactory; *S*, satisfactory; *I*, ideal.

A total of 87.5% (7/8) of “anaortic” configurations, compared with 25% (1/4) of “aortic” configurations, led to unsatisfactory grafts in at least one of the five patients (*P* = 0.038) (Figure 5). Composite “aortic” configuration A led to unsatisfactory grafting configurations in Patients 2 and 5. The other three “aortic” grafting configurations with three separate grafts and inflows (configurations B, C, and D) never led to an unsatisfactory grafting configuration [0% (0/15)]. “Anaortic” configurations with two inflows (LIMA and RIMA) (configurations E, F, I, J, L) led to unsatisfactory configurations in 80% of patients (4/5), 24% of the time (6/25). However, configuration J was satisfactory in all patients. Configurations with one inflow (configurations G, H, K) led to unsatisfactory configurations in all patients [100% (5/5)], 40% of the time (6/15).

### 3.4. Standard surgeon decision-making

There was a wide range of cardiac surgical experience among the participating surgeons (Table 8). The most common “aortic” grafting strategy selected with standard decision-making was configuration C (62.5%), whereas the most common “anaortic” strategy was configuration E (71.25%) and no surgeons in this study chose configurations G or L with very few selecting configurations I or K (Table 9). As few surgeons selected configuration A [15% (12/80)] which was the only “aortic” configuration that led to unsatisfactory grafting in two patients, standard decision-making led to very few unsatisfactory “aortic” grafting configurations [5% (4/80)] (Table 9, Figure 5). However, as most surgeons selected “anaortic” configuration E [71.25% (57/80)] which led to unsatisfactory grafting in one patient as well as “anaortic” configuration H [13.75% (11/80)] with unsatisfactory grafting in two patients the rate of unsatisfactory “anaortic” selections with standard decision-making was higher [21.25% (17/80)] (Table 9, Figure 5). An “aortic” configuration was preferred over an “anaortic” configuration, 77.5% (62/80) of the time. Half (8/16) of the surgeons chose at least one “anaortic” strategy among the five patients in preference to an “aortic” configuration.

**Table 8:** Experience of cardiac surgeons.

**Characteristic**	**Surgeons** **(*n* = 16)**
Number of years in practice (median, range)	8.5 (1–30)
Number of CABG operations in career (median, range)	875 (55–6,100)
Number of CABG operations per year (median, range)	75 (15–205)
Number of BIMA operations per year (median, range)	13.75 (0–110)
BIMA utilization rate per year (proportion, range)	427/1,365 (31.28%), (0–85%)
Number of surgeons performing more than 25 BIMA operations per year (proportion) (high volume)	4/16 (25%)
Number of surgeons performing < 1 BIMA operation per year (proportion)	3/16 (18.75%)
BIMA utilization rate in high volume BIMA surgeons (proportion, range)	315/590 (53.39%), (35.48–85%)
BIMA utilization rate in lower volume BIMA surgeons (proportion, range)	112/775 (14.45%), (0–25%)

BIMA, bilateral internal mammary arteries.

**Table 9:** Graft configuration selections with standard and computer informed strategy.

	**Standard** **(*n* = 80)**	**Computer** **(*n* = 80)**	***P-*value**
**Preferred aortic configuration**			
Configuration A	12/80 (15%)	9/80 (11.25%)	0.641
Configuration B	10/80 (12.5%)	13/80 (16.25%)	0.653
Configuration C	50/80 (62.5%)	47/80 (58.75%)	0.746
Configuration D	8/80 (10%)	11/80 (13.75%)	0.626
Totals	80/80 (100%)	80/80 (100%)	
**Preferred anaortic configuration**			
Configuration E	57/80 (71.25%)	41/80 (51.25%)	0.015
Configuration F	2/80 (2.5%)	17/80 (21.25%)	< 0.001
Configuration G	0/80 (0%)	0/80 (0%)	1
Configuration H	11/80 (13.75%)	8/80 (10%)	0.626
Configuration I	0/80 (0%)	2/80 (2.5%)	0.497
Configuration J	10/80 (12.5%)	10/80 (12.5%)	1
Configuration K	0/80 (0%)	2/80 (2.5%)	0.497
Configuration L	0/80 (0%)	0/80 (0%)	1
Totals	80/80 (100%)	80/80 (100%)	
**Unsatisfactory grafting configurations selected**			
Aortic	4/80 (5%)	0/80 (0%)	0.641
Anaortic	17/80 (21.25%)	1/80 (1.25%)	< 0.001
**Ideal grafting configurations selected**			
Aortic	52/80 (65%)	49/80 (61.25%)	0.743
Anaortic	5/80 (6.25%)	23/80 (28.75%)	< 0.001

### 3.5. Impact of computational models on surgeon decision-making

As standard surgeon decision-making led to the selection of fewer unsatisfactory “aortic” grafting configurations compared with “anaortic” configurations, the integration of the computational model-generated predictions by the cardiac surgeons led to a significant decrease in the selection of unsatisfactory grafting configurations for “anaortic” [21.25% (17/80) vs. 1.25% (1/80), *P* < 0.001] but not “aortic” techniques [5% (4/80) vs. 0% (0/80), *P* = 0.641] (Table 9). Similarly, there was an increase in the selection of ideal configurations for “anaortic” [6.25% (5/80) vs. 28.75% (23/80), *P* < 0.001] but not “aortic” techniques [65% (52/80) vs. 61.25% (49/80), *P* = 0.743] (Table 9).

For the primary outcome measure, the number of surgeons that changed from choosing at least one unsatisfactory grafting configuration to having no unsatisfactory grafting configurations was significant for “anaortic” configurations (13/16 = 81.25%, *P* < 0.001) but not for “aortic” configurations (2/16 = 12.5%, *P* = 0.480) (Table 10). The computational model predictions also led surgeons to make more patient-specific grafting selections for the “anaortic” configurations. The number of surgeons that changed from choosing no unique grafting configurations to having at least one unique grafting configuration across the five patients was significant (12/16 = 75%, *P* = 0.002) (Table 10). Half (8/16) of computer-informed surgeons used a configuration that they earlier stated they would not use and 56.25% (9/16) changed the order of their ranking preferences with the addition of myocardial perfusion data.

**Table 10:** Surgeon change in selection of unsatisfactory and unique grafting configuration.

	**Computer: unsatisfactory aortic grafting configurations (no.)**	**Computer: satisfactory aortic grafting configurations (no.)**
**Aortic**		
Standard: unsatisfactory aortic grafting configurations (no.)	0	2
Standard: satisfactory grafting configurations (no.)	0	14
*P* = 0.480		
	**Computer: unsatisfactory anaortic grafting configurations (no.)**	**Computer: satisfactory anaortic grafting configurations (no.)**
**Anaortic**		
Standard: unsatisfactory anaortic grafting configurations (no.)	1	13
Standard: satisfactory anaortic grafting configurations (no.)	0	2
*P* < 0.001		
**Aortic**		
	**Computer: unique aortic grafting configurations (no.)**	**Computer: no unique aortic grafting configurations (no.)**
Standard: unique aortic grafting configurations (no.)	1	3
Standard: no unique aortic grafting configurations (no.)	5	7
*P* = 0.724		
**Anaortic**		
	**Computer: unique anaortic grafting configurations (no.)**	**Computer: no unique anaortic grafting configurations (no.)**
Standard: unique anaortic grafting configurations (no.)	2	0
Standard: no unique anaortic grafting configurations (no.)	12	2
*P* = 0.002		

## 4. Discussion

### 4.1. Role for predictive computational flow modeling in surgical planning

After the degree of functional coronary stenosis, graft configuration has the most significant influence on graft and native coronary artery flows (10). Despite the tendency of surgeons for using a “one-size fits all” approach, hemodynamic predictions from this study suggest that surgeons need to tailor composite and sequential grafting configurations for each individual patient. Anaortic configurations based on a smaller number of separate inflows are more prone to steal of flow and competitive flow affecting upstream segments (17).

“Anaortic” configuration J, with its sequential arrangement, was favorable for Patients 1, 2, 3, and 4 as the RCA stenoses were equal to or greater than the CIRC stenoses (31). In Patient 5, although the OM vessel stenosis was at 99% and the RCA at 90% (iFR 0.73), the flows in the graft segment to PDA were still adequate at 15.29 ml/min. A higher iFR for the RCA stenosis in this patient could compromise configuration J as being universally satisfactory. This observation highlights that the complexity of determining unsatisfactory grafting configurations is potentially beyond the capabilities of using simple heuristics and may require the quantitative predictive value of computational modeling to determine the complex interplay between graft and host vessels and distal run-off.

Standard surgeon decision-making culminated in four composite Y-graft “aortic” selections [5% (4/80)] and 17 “anaortic” selections [21.25% (17/80)] which, on the basis of predictive hemodynamics, could lead to unsatisfactory grafts with poor patency. This result reveals a significant clinical opportunity for improvement in graft selection and function for both surgeons and patients given that options exist to avoid these configurations. In a clinical study of 120 patients undergoing complex composite grafting procedures using BIMA, the graft patency at a mean follow-up of 29.9 ± 33.1 months for arterial grafts was between 80 and 98.7% (32). Although grafts may appear to be patent initially, those with poor flow have been found to be occluded within 1 year (33, 34).

The computational model was effective in reducing the proportion of surgeons choosing unsatisfactory “anaortic” configurations from 87.5 to 6.25% (*P* < 0.001), far exceeding the learning change set *a priori* at 30% (28). One surgeon continued to pursue an unsatisfactory grafting configuration for one patient which may be explained by cognitive biases when evaluating computational models (35). The computational model influenced 50% of surgeons to select grafting configurations that they stated they would not usually use and led to more patient-specific tailoring of “anaortic” configurations, rather than a “one-size fits all” approach. This change in decision-making is understandable as the biophysics of the coronary circulation is complex, often difficult to completely measure and highly patient-specific. Thus, the standard decision-making for a CABG configuration is made in an environment of significant uncertainty (36). This study demonstrates that, when faced with uncertainty, the additional quantified flow information provided by predictive computational modeling will change surgical planning especially for configurations involving composite grafts.

The provision of additional myocardial perfusion data led 56.25% (9/16) of surgeons to change their graft configuration rankings. Indeed, surgeons tend to be poor at predicting qualitative effects on perfusion post CABG (37) and are not accustomed to assimilating data providing quantification of myocardial perfusion.

### 4.2. Limitations and future directions

There were a number of limitations in this study relating to how surgeons were engaged. Using only five patients did allow 12 grafting configurations to be interrogated on each patient. While relevant predictive hemodynamic data were presented to all 16 surgeons, other TTFM parameters which “COMCAB” is capable of measuring such as diastolic filling percentage and backward flow were not provided, to avoid information overload. This would not affect results as this study addressed situations of flow competition rather than technical anastomotic errors and hence providing MGF and PI were considered sufficient (38). Despite this, the study still typically took up to 45 min for each surgeon to complete. As this virtual surgical planning study omitted the real-life execution of CABG, the authenticity of surgeon responses may also be questioned. Only four out of 16 surgeons performed more than 25 BIMA operations per year and their rate of BIMA use was 53.39% (35.48–85%). For the other 12 surgeons, the BIMA use was only 14.45% (0–25%). This variability is consistent with 20% TAG utilization in Europe, up to 80% in some Australian centers (39) and 3% overall BIMA usage on multi-institutional analysis (40). A further subgroup analysis is planned as an extension to this study to investigate the effect of surgeon experience on engagement with information provided by the computational modeling. Future studies could also be streamlined by including more surgeons who perform anaortic total arterial OPCABG revascularisation using BIMA or even CABG involving more composite and sequential grafts.

The limitations arising from computational modeling have been described previously (25). With more invasive clinical patient data available, the idealized generic parameters of aortic root pressure, LV pressure, heart rate, CO, total arterial compliance and systemic arterial branch dimensions could be made more patient-specific. Despite this, the differences in patient-specific coronary artery disease resulted in differing outcomes from different grafting configurations between patients. The 7 h duration required to create the computer models included manual processing and therefore further automation of methods would be beneficial. Although hemodynamic predictions generally agreed with *in vivo* clinical data from other studies, further validation of the computational models could also be performed in the same patient data set by measuring *in vivo* pre-operative and post-operative iFR, intraoperative TTFM, as well as post-operative myocardial perfusion imaging.

The present study investigated 12 different TAG configurations using BIMA and RA for severe triple vessel coronary disease for each of five patients and is therefore the most comprehensive study of its kind in the literature, modeling 60 virtual grafting configurations (41–43). The computational methodology developed has the potential to investigate a wide range of other CABG configurations and hemodynamic scenarios. It is uncertain whether composite grafts can sustain adequate perfusion at both rest and hyperemia (44, 45). Future models could also account for variations in flow and incorporate the effects of cardiopulmonary bypass, anesthesia, exercise, hyperemia, coronary autoregulation and increased graft diameters over time. They could also investigate patients with saphenous vein grafts, poorer ejection fractions, or those with LV hypertrophy and microvascular coronary disease where the use of anaortic configurations with composite Y-grafts has been clinically cautioned (44).

Patient-specific computational modeling provides important predictive hemodynamic flow information that cardiac surgeons can incorporate into their decision-making when planning graft configurations for individual patients. Surgeons take heed of computer model predictions as their current practice involves significant uncertainty regarding native coronary and bypass graft flows achieved following CABG. Based on these results, “COMCAB” has the potential to be a promising clinical decision-support software tool for personalized surgical planning for CABG configurations. This is particularly important in avoiding situations of flow competition affecting bypass graft patency arising from the use of composite and sequential grafts such as those used more frequently in “anaortic” grafting techniques.

## Data availability statement

The original contributions presented in the study are included in the article/Supplementary material, further inquiries can be directed to the corresponding author.

## Ethics statement

The studies involving human participants were reviewed and approved by the Health and Disability Ethics Committee (HDEC) New Zealand (15/STH/245 and 29/03/2016). The patients/participants provided their written informed consent to participate in this study.

## Author contributions

KC and NS conceptualized the computational modeling. AP improved the code efficiency and visualization of the computational models. KC, PM, and NS conceptualized the study involving the cardiac surgeons. KC, AP, SW, PM, and NS edited the manuscript for intellectual content. All authors gave approval for the final version of the manuscript to be published.

## Supplementary material

Data Sheet 1 to 5 are each an excel file for each of the 5 patients containing 15 worksheets with segmented coronary geometry as well as vessel segment data and relations for non-diseased, stenotic, and 12 grafting configurations A to L.
